# Evidence of impaired neuromuscular responses in the support leg to a destabilizing swing phase perturbation in hemiparetic gait

**DOI:** 10.1007/s00221-016-4743-0

**Published:** 2016-08-05

**Authors:** Bahar Sharafi, Gilles Hoffmann, Andrew Q. Tan, Yasin Y. Dhaher

**Affiliations:** 1 Liberty Mutual Research Institute for Safety, 71 Frankland Road, Hopkinton, MA 01748 USA; 2 Department of Physical Medicine and Rehabilitation, Northwestern University, Chicago, IL USA; 3 Sensory Motor Performance Program, Rehabilitation Institute of Chicago, Chicago, IL USA; 4 Northwestern University Interdepartmental Neuroscience, Northwestern University, Chicago, IL USA; 5 Department of Biomedical Engineering, Northwestern University, Evanston, IL USA

**Keywords:** Hemiparetic gait, Long-latency reflex, Interlimb reflex, Bilateral impairment, Reactive balance control

## Abstract

The neuromuscular mechanisms that underlie post-stroke impairment in reactive balance control during gait are not fully understood. Previous research has described altered muscle activations in the paretic leg in response to postural perturbations from static positions. Additionally, attenuation of interlimb reflexes after stroke has been reported. Our goal was to characterize post-stroke changes to neuromuscular responses in the stance leg following a swing phase perturbation during gait. We hypothesized that, following a trip, altered timing, sequence, and magnitudes of perturbation-induced activations would emerge in the paretic and nonparetic support legs of stroke survivors compared to healthy control subjects. The swing foot was interrupted, while subjects walked on a treadmill. In healthy subjects, a sequence of perturbation-induced activations emerged in the contralateral stance leg with mean onset latencies of 87–147 ms. The earliest latencies occurred in the hamstrings and hip abductor and adductors. The hamstrings, the adductor magnus, and the gastrocnemius dominated the relative balance of perturbation-induced activations. The sequence and balance of activations were largely preserved after stroke. However, onset latencies were significantly delayed across most muscles in both paretic and nonparetic stance legs. The shortest latencies observed suggest the involvement of interlimb reflexes with supraspinal pathways. The preservation of the sequence and balance of activations may point to a centrally programmed postural response that is preserved after stroke, while post-stroke delays may suggest longer transmission times for interlimb reflexes.

## Introduction

Stroke survivors have significant residual deficits, related to balance control, as evidenced by a high rate of falls (Weerdesteyn et al. 2008). Walking in the real world often requires corrective reactions to recover or maintain balance in response to unexpected perturbations. Given that falls in stroke survivors most often occur while walking (Hyndman et al. 2002), reactive balance control during gait is critical to stroke survivors’ ability to walk independently. Previous reports of post-stroke neuromuscular responses to postural perturbation have focused on balance recovery from static postures (e.g., Marigold and Eng 2006; Kirker et al. 2000). While decreased stability in response to gait perturbations has been documented after stroke (Krasovsky et al. 2013), investigation of the neuromuscular responses during the functional task of balance recovery while walking is lacking in this population. The goal of this work was to examine post-stroke deficits in the functional, neuromuscular responses that contribute to reactive balance control during gait.

Emerging evidence suggests that chronic motor control impairments in post-stroke individuals involve abnormal multi-segmental behaviors. For example, abnormal coupling of stretch reflexes has been quantified under isometric conditions between hip adductor and knee extensor muscles in paretic legs of stroke survivors (Finley et al. 2008). We propose that, given the complex, bi-planar nature of balance control during gait, under destabilizing conditions, aberrant multi-joint neural constraints may impede the ability to generate appropriate neuromuscular responses.

To examine neuromuscular responses contributing to balance recovery during gait, we employed an induced trip. A trip involves interrupting the swing leg, necessitating a stabilizing neuromuscular response in the support leg (the stance leg contralateral to the perturbation). Immediately following a trip, prior to foot contact of the perturbed leg, the role of the support leg is to stabilize the body by arresting the angular momentum, resulting from the trip, and to provide time and clearance for the placement of the perturbed foot (Pijnappels et al. 2004). The focus of this study was to characterize the neuromuscular responses to a trip in the support leg. The initial single support phase of stabilization provides the opportunity to examine the stabilizing neuromuscular response in the paretic and nonparetic support legs in the absence of compensation from the opposite leg.

Previous studies have reported post-stroke delays of muscle activations in the paretic leg following postural perturbations from standing position (Marigold and Eng 2006; Kirker et al. 2000; Dietz and Berger 1984). During gait, bilateral delays were observed in an obstacle avoidance experimental paradigm (van Swigchem et al. 2013). Additionally, bilateral attenuation of interlimb cutaneous reflexes has been reported during gait (Zehr and Loadman 2012). While the origin of these post-stroke changes is unclear, examination under isolated single-joint constraints pointed to stroke-mediated impairments in interlimb spinal pathways (Stubbs et al. 2012). In light of these findings, and given the proposed role of interlimb reflexes in stumble correction during gait (e.g., Haridas et al. 2005), we hypothesized that altered timing, sequence, and magnitudes of perturbation-induced muscle activations would emerge in paretic and nonparetic support legs of stroke survivors compared to healthy control subjects.

## Methods

### Subjects

Eleven stroke survivors and eight healthy control subjects (Table 1) completed this study. Stroke survivors were recruited if they had a unilateral stroke at least 6 months prior to the test, hemiparesis of the lower limb, no cognitive deficits that would prevent them from performing the experimental protocol, and no prior history of injury or surgery to the lower limbs. All participants had to be able to walk for at least 10 min without wearing a brace, assistive device, body weight support, or reliance on treadmill handrails. Experimental procedures were approved by the Institutional Review Board of Northwestern University and complied with the principles of the Declaration of Helsinki. Informed consent was obtained prior to testing from all participants.

**Table 1:** Subject information

Subject	Age (years)	Sex	Height (cm)	Weight (kg)	Lesion side	Months post-stroke	Stroke type^a^	Berg (56)	LMFM^a^ (34)	SPFM^a^ (24)	MMSE^a^ (30)	Speed (m/s)
S1	52	F	162	63	L	35	H	51	18		30	0.3
S2	53	F	168	66	L	36	H	49	24			0.6
S3	58	M	180	78	R	55	I	49	22	24	21	0.6
S4	58	M	188	90	L	75	I	51	22	24		0.6
S5	65	F	157	57	L	117	I	41	17	24		0.5
S6	58	M	185	92	L	102	I	45	22		30	0.5
S7	57	M	180	107	R	76	H	54	26	24	30	0.9
S8	57	M	173	83	L	33		50	24	24	29	0.9
S9	48	M	165	93	R	184		46	22	18	29	0.4
S10	42	M	175	84	L	82		56	26	24	28	1.1
S11	55	M	173	84	L	15	I	49	16		29	0.6
C1	51	M	183									1.3
C2	52	M	183									1.1
C3	51	M	175									1.3
C4	44	M	178									1.4
C5	46	F	152									1.2
C6	51	F	173									1.4
C7	59	F	168									1.1
C8	41	F	152									1.3

Perfect scores are shown in parentheses

*S* post-stroke subject identifiers, *C* control subject identifiers, *H* hemorrhagic, *I* ischemic, *LMFM* Fugl-Meyer (lower limb motor), *SPFM* Fugl-Meyer (sensation and proprioception), *MMSE* mini-mental state evaluation

^a^Missing entries indicate unavailable values. Speed is self-selected fast walking speed

### Experimental setup and protocol

**Fig. 1:**
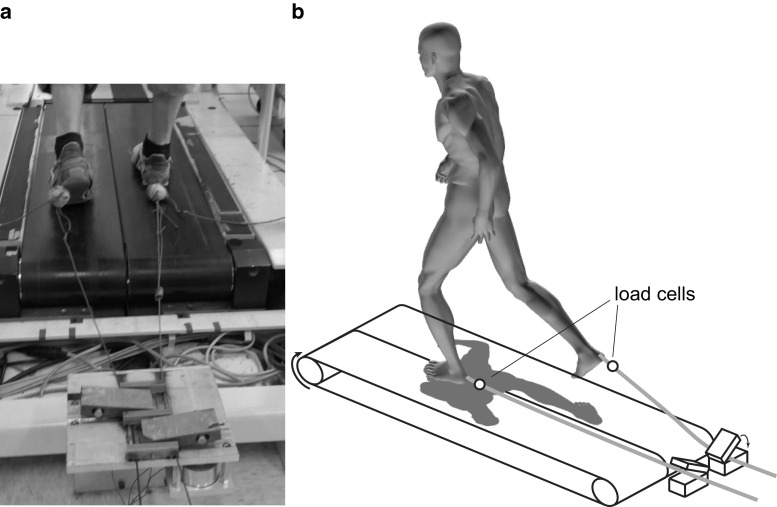
Subjects walked on an instrumented split-belt treadmill. Trips where induced by catching and holding the retractable cable attached to either foot during swing phase using a custom-made device. Photograph (**a**) and schematic (**b**) of the setup are shown

Participants walked on an instrumented split-belt treadmill (Tecmachine, Andrézieux Bouthéon, France) at their self-selected fast speed. Subjects were instructed to walk at the fastest speed that they could maintain for continuous intervals, each lasting at least 10 min, and in total adding up to at least 1 h. This allowed the satisfaction of two criteria: (1) walking conditions were natural to each subject and (2) walking speed was fast enough that the interruption of the swing foot was challenging to the maintenance of balance. A custom code calculated the average swing phase duration for each subject based on the vertical ground reaction forces output by the treadmill force plates. A custom device (Shirota et al. 2014) induced trips by interrupting the foot via a retractable cable attached to the foot (Fig. 1). There was minimum slack in the cables while subjects walked so as not to get caught under the feet. To interrupt the foot, a solenoid valve closed a flap over the cable attached to the foot for 150 ms. Each cable had a uniaxial load cell to measure the trip force (force required to interrupt the foot). Closing of the solenoid valves was timed so that trip onset, defined as the moment at which trip force exceeded 10 N, occurred between 10 and 30 % of swing. Early swing phase was chosen for the perturbation to allow for a longer single support phase during the initial phase of stabilization.

To minimize anticipation of the trip, perturbations were applied to both feet in random order and at random intervals, at least 1 min apart, and participants were engaged in conversation while they walked. The length of each recorded trial was 10 s and included at least two unperturbed strides prior to the trip. Participants wore a safety harness to prevent falling in case of unsuccessful recovery. The harness cable had sufficient slack so as not to interfere unless a fall was imminent. Participants were allowed to grab the treadmill handrails in case of a fall; however, they were advised to avoid touching the handrails unless absolutely necessary following a trip.

### Data collection

Gait kinematics were recorded using an eight-camera motion capture system (Motion Analysis Corporation, Santa Rosa, CA, USA). Forty-nine reflective markers were placed on each participant. Marker trajectories were recorded at 100 Hz. Surface EMG (Delsys, Boston, MA, USA) was recorded bilaterally from the soleus (SOL), medial gastrocnemius (MGAS), peroneus longus (PL), tibialis anterior (TA), semitendinosus (ST), biceps femoris (BF), vastus medialis (VM), vastus lateralis (VL), rectus femoris (RF), adductor magnus (ADD), gluteus maximus (GMA), and gluteus medius (GME). Ground reaction forces, trip force, harness force, and EMG were sampled at 1000 Hz. The raw EMG signal from each muscle was high-pass filtered at 250 Hz, rectified, then low-pass filtered at 100 Hz with a zero-phase fourth-order Butterworth filter. Trip force and ground reaction forces were filtered at 30 and 15 Hz, respectively. All trials were recorded on video to visually determine the incidence of handrail use. A trial was categorized as a failed recovery attempt if the harness was engaged with a load greater than 10 % of body weight or if the participant was unable to recover without grabbing the handrail.

### Kinematic onsets

Onset latencies of kinematic events from trip onset were determined as follows. Joint angle trajectories, output by Cortex system (Motion Analysis Corporation, Santa Rosa, CA, USA), were low-pass filtered at 10 Hz using a fourth-order Butterworth filter. Mean profiles were calculated from all unperturbed strides and subtracted from individual perturbed strides. To establish a baseline level and a baseline level of variability, five data points (50 ms) of the subtracted profile, prior to perturbation onset, were used to calculate a mean and SD. An onset of deviation was detected if the subtracted value, averaged over four data points (40 ms), was more than 4 SD away from the baseline level.

### EMG onsets

EMG onset latencies were calculated relative to trip force onset. Alternatively, latencies could have been reported relative to the earliest detected onset of joint angle deviation, i.e., ankle dorsiflexion. Given the similarity of ankle perturbation onsets across groups, this method would not have altered our findings (results). EMG time histories from all trials were conditioned using the Teager–Kaiser energy operator (Solnik et al. 2010). For each subject and each muscle, EMG mean and SD were calculated for all unperturbed strides aligned from heel strike. The mean unperturbed EMG was subtracted from individual perturbed strides aligned from the heel strike of the support leg. A perturbation-induced onset was determined if the subtracted, conditioned EMG, averaged over 20 ms, exceeded 4 SD of the conditioned EMG at the corresponding point in the unperturbed gait cycle. This threshold was based on a sensitivity analysis of a subset of post-stroke and healthy EMG time series and was chosen to maximize agreement with expert visual detection and minimize the number of false positives. Onset latencies detected earlier than 30 ms from trip onset were designated as false positives, given that neuromuscular latencies in the contralateral leg cannot be shorter than a monosynaptic stretch reflex.

### Similarity of EMG onset sequence

For each support leg condition (control, nonparetic, and paretic), we defined the mean sequence of perturbation-induced activations as a vector, *V*_12×1_, containing the respective mean onset latencies. To evaluate the similarity between the mean sequence in the paretic and nonparetic support legs with that in the control support leg, we calculated the Euclidean distance, *d*, between this vector, for each condition, with that of the control condition, *V*_c_, as follows: $d_{\text{np}} = \sqrt {\left( {V_{\text{np}} - V_{\text{c}} } \right)^{\text{T}} \cdot \left( {V_{\text{np}} - V_{\text{c}} } \right)}$ and $d_{n} = \sqrt {\left( {V_{\text{p}} - V_{\text{c}} } \right)^{\text{T}} \cdot \left( {V_{\text{p}} - V_{\text{c}} } \right)}$, where the subscript np denotes nonparetic and p denotes paretic. If *d* was significantly smaller than the distance expected if the sequence was generated at random (e.g., d’Avella and Bizzi 2005), we concluded that the mean sequence of perturbation-induced activations for that support leg condition (paretic or nonparetic) was similar to the mean control sequence. To perform this comparison, we generated a set of 10^4^ vectors, $V_{\text{random}}^{(i)}$, by randomly permutating the elements of *V*. We calculated the Euclidean distance, $d_{\text{random}}^{(i)}$, between each $V_{\text{random}}^{(i)}$ and the mean control sequence, *V*_c_. From a 100 bin histogram of $d_{\text{random}}^{(i)}$ values, we generated an empirical probability density function of distances of randomly generated onset sequences for each limb condition. We determined the *p* value for rejecting the null hypothesis that the actual distance, *d*, was drawn from this distribution by integrating the empirical probability density function from 0 to *d*.

### EMG magnitudes

To determine the magnitude of perturbation-induced activations across muscles as a function of time after perturbation, we subtracted the mean of band-passed, rectified, and low-passed EMG of unperturbed stance phase from the mean EMG of the support leg during perturbed trials. To enable comparison across subjects, the subtracted EMG was normalized by the peak of mean EMG of unperturbed gait cycles. Normalized, subtracted EMG profiles were averaged within groups. Within-group average time series were then time-averaged by binning a 180-ms post-perturbation interval into 20 ms bins. Onset and magnitude data for a given support leg condition were only presented for muscles that activated in more than of 50 % of trips across all subjects.

### Between-group comparisons

Comparisons across support leg conditions (control, nonparetic, and paretic) were made using repeated-measures mixed-effect models (Littell et al. 1998). The value from each trial was considered a repeated measure associated with the corresponding subject. A compound symmetry correlation structure was used to take into account correlations within each subject. All statistical analyses were performed in SAS 9.2 (SAS Institute Inc., Cary, NC, USA).

### Analyses of confounding effects

The following variables were examined as covariates (“Appendix”): walking speed, perturbed ankle stiffness, and support leg posture at trip onset.

## Results

Post-stroke and control subjects walked at average speeds of 0.64 ± 0.07 m/s (mean ± SE) and 1.26 ± 0.04 m/s, respectively. Between eight and twenty-eight trips (16 mean ± 5 SD) were successfully recorded for each leg. Perturbations of the paretic swing leg were successfully recorded in 10 of the 11 post-stroke subjects. A total of 128, 164, and 159 perturbations of control, nonparetic, and paretic swing legs were successfully recorded. Mean trip onsets and trip forces are presented in Table 2. Of the total number of perturbed trials for the control, nonparetic, and paretic swing legs (corresponding to control, paretic, and nonparetic support legs), 26, 68, and 33 %, respectively, resulted in failed recovery attempts.

**Table 2:** Trip onset, force, perturbed ankle stiffness, and swing leg kinematic onsets for perturbations applied to control, nonparetic, and paretic swing legs

	Swing leg			*p* values		
	Control	Nonparetic	Paretic	Control versus nonparetic	Control versus paretic	Nonparetic versus paretic
Peak trip force	203.8 ± 9.8	146.5 ± 7.5	107.8 ± 18.8	<0.001	<0.001	0.014
Trip onset (% of average swing duration)	25.8 ± 0.6	24.0 ± 1.0	20.8 ± 1.4		0.006	0.03
Ankle stiffness (Nm/°)	0.66 ± 0.03	0.76 ± 0.12	1.37 ± 0.57			
Onset ankle dorsiflexion (ms)	27.1 ± 0.4	25.3 ± 0.5	24.7 ± 0.7			
Onset knee flexion (ms)	86.5 ± 3.0	77.3 ± 1.8	83.9 ± 3.2			
Onset hip extension (ms)	64.2 ± 1.8	74.3 ± 1.0	102.5 ± 3.0		<0.001	0.03

Trip onset was defined as the moment at which trip force exceeded 10 N. Values are intragroup mean ± SE of the mean. Only *p* values that show significant differences between support leg conditions (*p* < 0.05) have been presented

### Onsets of kinematic events

**Fig. 2:**
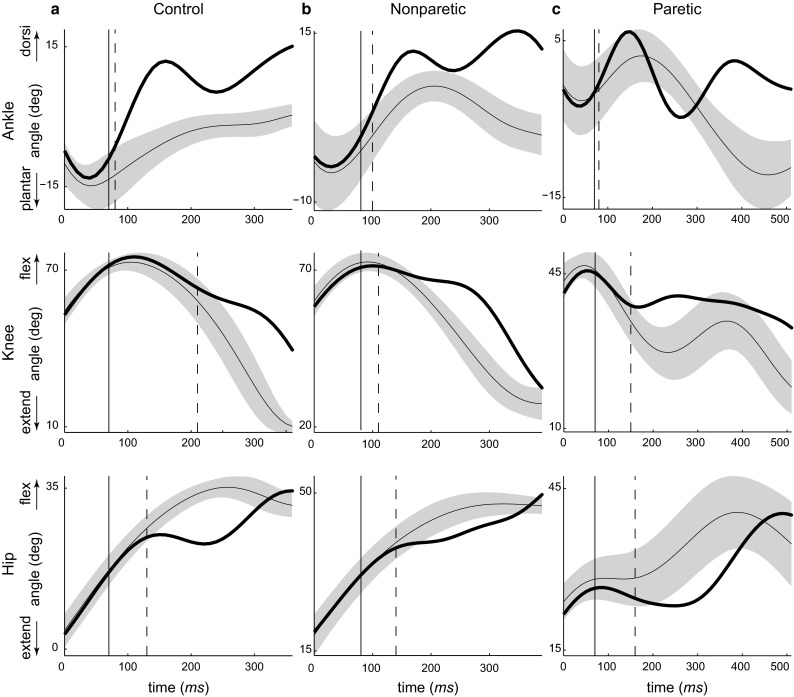
Representative swing phase kinematics are shown from a control subject (**a**) and a stroke survivor (**b**, **c**). The *thin solid curve* and *shaded area* represent the mean and 2 SD of unperturbed swing phase trajectory. The *thick solid curve* depicts the joint trajectories of the perturbed swing leg in one trip trial. *Solid vertical line* denotes trip onset. *Dashed vertical line* denotes the detected onset of joint angle deviation

**Fig. 3:**
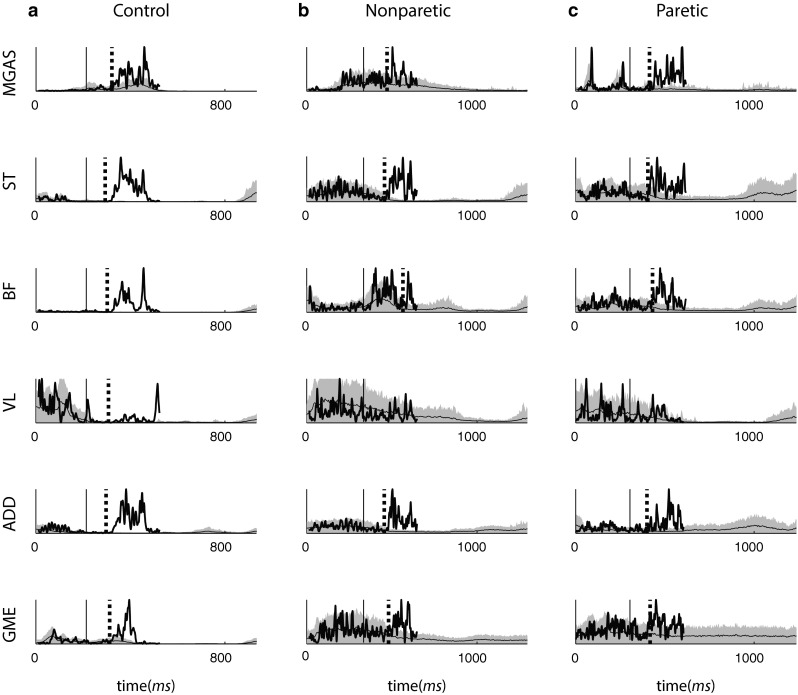
Representative support leg rectified and filtered EMG time series are shown from a number of muscles for a control subject (**a**) and a stroke survivor (**b**, **c**). The *thin solid curve* and *shaded area* represent the mean and 2 SD of unperturbed EMG. The *thick solid curve* depicts the EMG of the support leg in one trip trial. *Solid vertical line* denotes trip onset. *Dashed vertical line* denotes the detected onset of perturbation-induced EMG activity in the muscle. Time zero is the heel strike of the support leg. The *vertical axis* is in mV and is not on the same scale for all plots

The interruption of the swing foot induced a deviation of swing leg joint angles from their unperturbed trajectory. The perturbation increased the dorsiflexion of the ankle and impeded the extension and flexion of the knee and hip, respectively. Perturbation-induced onsets of joint angle deviations in control, nonparetic, and paretic swing legs are given in Table 2. No significant differences were found between limb conditions in the onset of deviations of the ankle and knee flexion angles. The onset of hip extension was significantly later in the paretic perturbed hip compared to nonparetic and control conditions. Representative swing leg kinematics and support leg EMG are shown in Figs. 2 and 3, respectively.

### EMG onsets

**Fig. 4:**
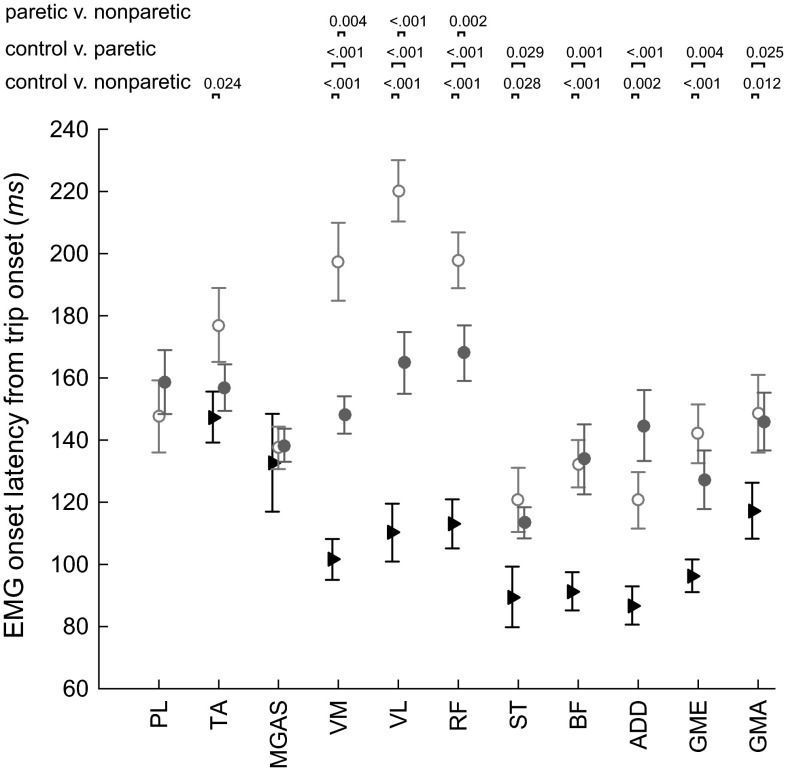
Mean EMG onset latencies were calculated for each group from trip onset. *Error bars* represent SE. *p* values smaller than 0.05 are shown for comparison of EMG onset latencies between control and nonparetic, control and paretic, and paretic and nonparetic support legs. In each group, only muscles that activated in more than an average of 50 % of trip trials across members of that group are shown. For example, the soleus was activated in an average of 43, 47, and 48 % of trials in the control, nonparetic, and paretic support legs, respectively

In control subjects, the earliest EMG onsets in the support leg occurred in the hamstrings (BF and ST), the hip adductor (ADD), and abductor (GME), with intragroup mean latencies of 87–96 ms from trip onset. These were followed by responses in the quadriceps (VM, VL, and RF) with mean latencies of 101–113 ms, the GMA (mean latency of 117 ms), and the MGAS (mean latency of 119 ms). The TA had a mean latency of 147 ms. The SOL and the PL were activated in less than 50 % of trips across control subjects. In paretic and nonparetic support legs, the earliest onsets appeared in the BF, ST, ADD, and GME muscles. However, the mean onset latencies of these muscles were delayed by 20–50 ms in the paretic leg. All proximal muscles (hamstrings, quadriceps, gluteals, and adductor magnus) had significantly delayed onsets in both paretic and nonparetic legs compared to control subjects. Significant differences between paretic and nonparetic legs were only found in the quadriceps, with longer onset latencies in the nonparetic quadriceps compared to the paretic (Fig. 4).

### Similarity of EMG onset sequence

The paretic support leg expressed a similar sequence of activations to the control support leg. We rejected the null hypothesis that *d*_p_, the Euclidean distance between the mean paretic sequence and the mean control sequence, was drawn from the distribution of randomly generated sequences (*p* = 0.014). In other words, the mean paretic sequence was significantly closer to the mean control sequence than can be expected if the paretic onset latencies were randomly ordered between muscles. In the nonparetic leg, although the distance with the control sequence, *d*_np_, was smaller than the mean distance expected at random, the null hypothesis could not be rejected (*p* = 0.28). This discrepancy arose from the long delays in the onsets of the nonparetic quadriceps. In fact, once quadriceps were eliminated from the analysis, the sequence of activations in the nonparetic support leg was significantly similar to the control support leg (*p* = 0.006).

### EMG magnitudes

**Fig. 5:**
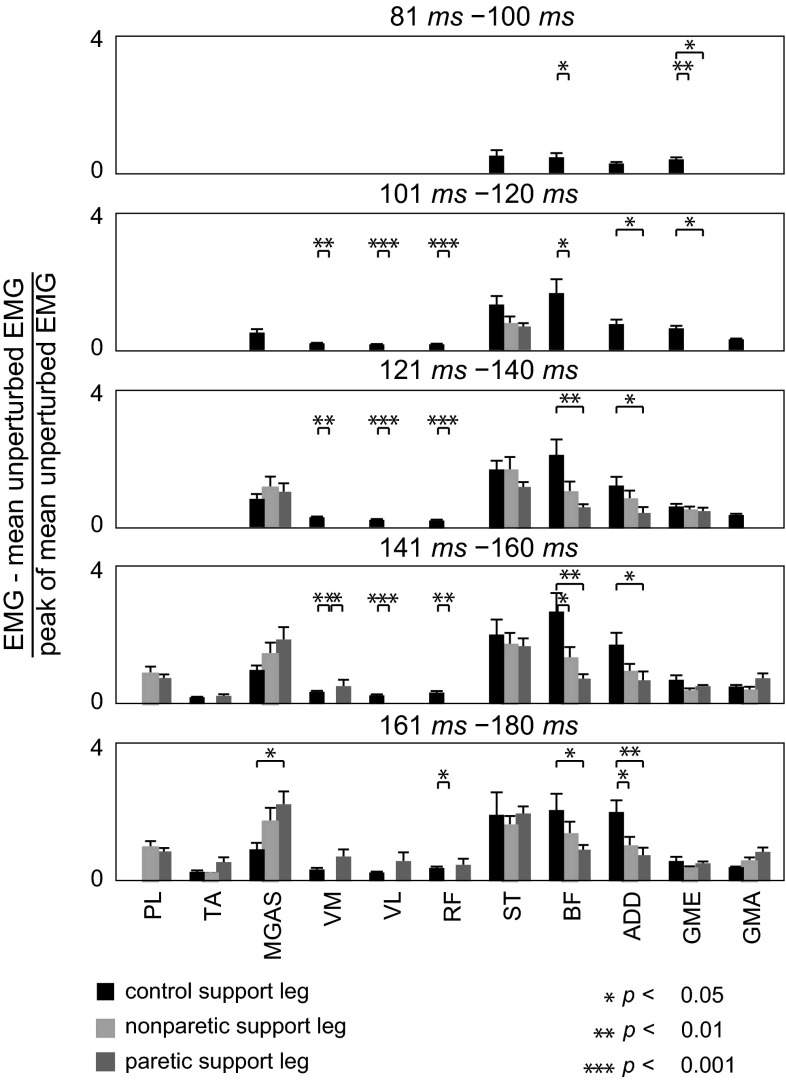
Mean EMG of unperturbed stance was subtracted from mean EMG of trip strides for the support leg and normalized by the peak mean EMG of unperturbed gait cycles for each subject. Group mean subtracted and normalized EMG was averaged for 20-ms intervals following trip onset for a 180-ms period. Nonzero mean magnitudes are shown only in intervals greater than the group mean onset latency of each muscle (Fig. 4). *Error bars* represent SE

Following the trip, the relative balance of magnitudes of perturbation-induced muscle activations in the support leg was dominated by the ST, BF, ADD, and MGAS, across all groups (Fig. 5). Magnitudes of normalized perturbation-induced activations were larger in the control ST, BF, ADD, and GME compared to the paretic and nonparetic legs (Fig. 5). Magnitudes of normalized perturbation-induced activations were larger in the nonparetic ST, BF, and ADD compared to the paretic leg, although significance was not reached.

## Discussion

The goal of this study was to determine whether timing, sequence, and balance of perturbation-induced muscle activations, in response to a unilateral gait perturbation, were altered in the paretic and nonparetic support legs of stroke survivors relative to healthy subjects. We found a largely similar sequence and balance of activations across paretic, nonparetic, and control support legs. The earliest perturbation-induced responses in the support leg appeared in the hamstrings and hip adductor and abductors. These muscles along with the gastrocnemius dominated the relative balance of activations. However, onsets of neuromuscular responses in the support leg were significantly delayed in stroke survivors compared to healthy control subjects, irrespective of whether the paretic or the nonparetic leg was perturbed.

Onset latencies reported here for control subjects were generally comparable to previous reports in ST, BF, RF, and VL, in a limited number of studies (Eng et al. 1994; Schillings et al. 2000; Pijnappels et al. 2005a). However, the onset latency of MGAS was significantly longer compared to these reports (Eng et al. 1994; Pijnappels et al. 2005a). Key differences in the experimental paradigm may have contributed to this discrepancy: (1) In the present study, the foot was interrupted using a flexible cable that decelerated the foot within an approximately 40-ms interval from trip onset, whereas the impact of the foot with a relatively rigid obstacle, as in the above studies, would have instantaneously stopped the forward movement of the foot. (2) In the above studies, the impact of the foot with an obstacle, in early swing phase, would have plantarflexed the ankle. In this study, the imposed perturbation dorsiflexed the swing ankle. It is possible that intralimb and interlimb reflexes, triggered by the perturbation imposed at the ankle, are direction-dependent. For instance, although we found no such comparison in healthy individuals, in spinal cord injured patients, the nature of ipsilateral heteronymous reflexes was strongly dependent on whether a dorsiflexion or a plantarflexion rotation was imposed at the ankle (Schmit et al. 2000). Given that early responses in the support leg can be largely attributed to the perturbation experienced by the swing leg (see “Nature of the observed responses”, below), it is likely that these differences in the applied perturbation resulted in differences in the timing of support leg neuromuscular responses.

### Nature of the observed responses

While we did not aim to directly address the underlying pathways mediating the responses, we made the following observations. The onset latencies of responses in the control group, with the possible exception of the TA, were below reported thresholds for voluntary reactions (Swinnen et al. 1995), suggesting the automatic nature of responses. It has been proposed that latencies of these orders of magnitude reflect the involvement of polysynaptic supraspinal pathways (Nielsen et al. 1997). A recent examination of interlimb reflexes following unilateral perturbation confirmed the contribution of a transcortical loop to observed responses (mean latency of 76 ms) (Stevenson et al. 2013).

The exact mechanisms contributing to the contralateral leg response may be multifactorial, including muscle afferents at the joints of the perturbed swing leg, local stretch reflexes at the joints of the support leg, vestibular postural reflexes, and cutaneous afferents from the support foot. The earliest detectable deviation in support leg joint angles appeared later than the onset of muscle activity in that leg. Therefore, it is unlikely that the observed response in the support leg was due to muscle afferents in the same leg. Moreover, EMG onsets preceded any detected deviation in the acceleration of the head or changes in the trajectory of the center of pressure, precluding the involvement of vestibular postural reflexes and cutaneous afferents from the support foot, in the early part of the response. Taken together, these observations suggest that the reflex responses in the support leg may have been triggered by muscle afferents from the perturbed leg. Muscle afferents at any of the joints of the perturbed leg may have contributed to neuromuscular responses in the support leg. Given the latencies of crossed reflexes, especially with the possible involvement of transcortical pathways (Stevenson et al. 2013), it is likely that the reported onsets, in a majority of muscles, were triggered due to muscle afferents at the swing ankle rather than the hip and knee joints.

### Preserved sequence and balance of perturbation-induced EMG after stroke

The sequence and balance of activation in the paretic and nonparetic support legs were largely consistent with those observed in control subjects. One might argue that the activation of the hamstrings and the hip adductor and abductors may comprise a centrally programmed activation synergy, forming a “first line of defense” on the support side. Our results suggest that this first line of defense is largely preserved following stroke. It has been suggested that postural responses are centrally programmed as a set of synergies, or activation patterns, with the selection of the appropriate pattern triggered by afferent input (Dietz 1992). This argument is supported by feline models that reveal the existence of a centrally coordinated postural response involving the reticular formation (Stapley and Drew 2009). It is conceivable that similar pathways contribute to the responses observed in humans.

### Bilateral delays in reflex onsets after stroke

Onsets of neuromuscular responses in the support leg were significantly delayed in a majority of muscles in stroke patients relative to control subjects. These onsets were similar between paretic and nonparetic support legs, with significant differences detected only in the quadriceps. Bilateral post-stroke impairment in the modulation of interlimb reflexes has previously been reported in response to cutaneous nerve stimulation (Stubbs et al. 2012; Zehr and Loadman 2012). Additionally, bilateral delays were reported in onsets of muscle activations when performing within-step adjustments (van Swigchem et al. 2013). This study characterized the effect of stroke on the timing and magnitudes of neuromuscular responses in the context of the functional task of balance recovery during gait. In response to perturbations from standing, delayed responses were only reported in the paretic leg contralateral to a perturbation (Dietz and Berger 1984). The presence of bilateral delays during gait may be due to the different nature of balance control during gait compared to standing. Following a perturbation from standing position, muscle activations follow a distal-to-proximal sequence and are limited to muscles acting in the plane of the perturbation. During gait, the reactive control of balance requires the cross-planar activation of muscles and involves a proximal-to-distal sequence. It is possible that different neural circuitries are engaged in the control of posture during gait compared to standing.

### Delayed knee stabilization in the nonparetic leg

Onset latencies of the quadriceps were significantly longer in the nonparetic compared to the paretic support leg. It could be argued that this asymmetry resulted from differences between afferent inputs from the perturbed paretic and nonparetic legs. If this were the case, we would expect this asymmetry to emerge across all muscles. Alternatively, one might argue that the asymmetrical latencies of the quadriceps are related to differences in the kinematics of the perturbed paretic and nonparetic swing legs. Onsets of perturbation-induced deviation were similar for nonparetic and paretic swing knees. However, while the nonparetic knee continued to extend, the paretic knee went into flexion (Fig. 2). Given the longer latencies of the quadriceps in the stroke group, differences in perturbation-induced knee kinematics between the paretic and nonparetic sides may have contributed to the difference in latencies of these muscles.

### Functional role of the observed responses

Our results extend the sagittal plane findings reported in healthy subjects (Pijnappels et al. 2005b; Eng et al. 1994; Schillings et al. 2000). We found that the hip adductors and abductors, along with the hamstrings, had the shortest response latencies and dominated the relative balance of activations during the early recovery period. This finding suggests that the motor control system prioritizes frontal plane stabilization, even in response to sagittal plane perturbations.

### Functional and clinical implications

Longer reflex latencies may be a limiting factor for stroke survivors’ ability to avoid falls in response to gait perturbations. While recovery from a trip can occur over multiple steps and is dependent on responses in both legs, delayed early responses in the support leg may result in excessive angular momentum and the inability to appropriately place the perturbed foot, thereby limiting the ability to arrest the forward directed angular momentum in subsequent steps.

Conversely, the cross-planar sequence and balance of perturbation-induced activations in stroke survivors were similar to healthy control subjects. The timing of post-stroke standing postural reflexes was shown to be modifiable (Marigold et al. 2005). Further studies are needed to determine whether improvements in response latencies may also be achieved under dynamic conditions. Finally, bilateral delays in the neuromuscular response measured in the present study support the argument that there is no unaffected side after stroke and highlight the importance of focusing rehabilitation efforts on both limbs.

### Limitations and confounding effects

We sought to examine functional responses to a destabilizing perturbation under natural walking conditions; therefore, subjects were instructed to walk at their self-selected fast speed. This meant that stroke survivors walked at speeds significantly slower than control subjects, potentially confounding the across-group comparisons. However, we found no significant effect of walking speed on onset latencies (“Appendix”). This finding was consistent with earlier literature. While changes in reflex size were observed with walking speed (Sinkjaer et al. 1996), no speed dependence was reported for the latencies of short-, medium-, or long-latency reflexes (Sinkjaer et al. 1996, 1999; Cronin et al. 2009; Grey et al. 2001). Additionally, it could be argued that, in the current experiment, differences in walking speed resulted in differences in perturbation velocity, which may have altered onset latencies. However, previous studies did not find onset latencies to be dependent on perturbation velocity (e.g., Cronin et al. 2009; Finley et al. 2013).

Characteristic differences between the populations in limb posture, joint stiffness, and background activation could also potentially confound the results. It could be argued that delayed EMG onsets in stroke survivors compared to control subjects may be accounted for by differences in these variables. However, we found no significant effect of support leg posture and perturbed ankle stiffness on onset latencies (“Appendix”). Additionally, examination of the literature yielded no evidence that onset delays, of magnitudes observed here, could be attributed to differences in background activation. A number of studies have shown no change in onset latency as a function of background activation for short-, medium-, and long-latency stretch reflexes in single-joint constrained experiments (Toft et al. 1991, 1989; Finley et al. 2012; Kimura et al. 2003). While some decrease in peak or onset latency has been reported with several-fold increases in background activation (Toft et al. 1989; Ogiso et al. 2002), these changes were much smaller (1–9 ms) than differences between stroke and control groups (20–60 ms). Taken together, these reports combined with the result of our analysis of the effect of covariates suggest that post-stroke onset delays observed in this study did not arise from differences in the above confounding effects. Rather, onset delays likely had neurophysiological origins.

Finally, the goal of this study was to examine changes in post-stroke neuromuscular responses in the stabilizing support leg. To examine the underlying mechanisms of these changes, statistical analyses of the relationship of joint kinematics and EMG in the perturbed leg to the neuromuscular responses in the support leg will be informative. In addition, the duration of the perturbation-induced response and the presence of co-activation are some factors, other than onset delays, that could contribute to impaired balance recovery. These factors will be examined in future investigations.

## Appendix: Analyses of confounding effects

We examined the effect of walking speed, support leg posture, and perturbed ankle stiffness, by including these variables as covariates in repeated-measures mixed-effect models, alongside support leg condition (control, nonparetic, and paretic). Each variable was first separately included in the model along with support leg condition. We examined: (1) whether the variable in question had a significant effect (*p* < 0.05) on onset latencies, and (2) whether with the inclusion of the covariate, limb condition remained a significant effect. If a covariate did not have a significant effect, we concluded that it did not contribute to differences in onset latencies, and it was excluded from subsequent analyses. If support leg condition remained a significant effect after including other covariates, we concluded that the difference between onset latencies across limb conditions could not be accounted for by these confounding effects.

### Walking speed

Across all support leg conditions and all muscles, when walking speed was included as a covariate alongside support leg condition in a repeated-measures mixed-effect analysis of variance, walking speed did not have a significant effect (*p* = 0.54) on onset latency, while support leg condition (paretic vs. control, and nonparetic vs. control) remained significant (*p* < 0.001). Walking speed was therefore excluded from the remaining analyses.

### Perturbed ankle stiffness

Perturbed ankle stiffness was estimated using the deviation of the sagittal plane ankle trajectory from unperturbed conditions within 10 ms of trip onset and an estimate of the trip force moment about the ankle. The force vector direction was along the trip cable. An estimate of the trip cable line of action was obtained using the origin of the cable on the device and a marker on the back of the heel, where the cable was attached. The moment arm of the trip force about the ankle, *l*, was calculated as the vertical distance between the ankle joint center and the cable line of action. The moment of the trip force was $T_{\text{trip}} = l \cdot F_{\text{trip}}$, where *F*_trip_ was the measured trip force. Ankle stiffness, *K*_ankle_, was calculated as follows: $K_{\text{ankle}} = {\text{d}}T_{\text{trip}} /{\text{d}}D_{\text{ankle}}$, where ${\text{d}}T_{\text{trip}} = T_{\text{trip}} \left( {{\text{trip}}\;{\text{onset}} + 10\;{\text{ms}}} \right) - T_{\text{trip}} \left( {{\text{trip}}\;{\text{onset}}} \right)$, *D*_ankle_ was the deviation in ankle trajectory calculated as the perturbed ankle trajectory less the average unperturbed trajectory, and ${\text{d}}D_{\text{ankle}} = D_{\text{ankle}} \left( {{\text{trip}}\;{\text{onset}} + 10\;{\text{ms}}} \right) - D_{\text{ankle}} \left( {{\text{trip}}\;{\text{onset}}} \right)$.

Estimated values of ankle stiffness are provided in Table 2. These values were within the reported range of values measured for intrinsic stiffness for control, nonparetic, and paretic ankles (e.g., Thajchayapong et al. 2006; Galiana et al. 2005). Across all support leg condition and all muscles, when ankle stiffness and support leg condition were included, ankle stiffness was not a significant effect (*p* = 0.16) on onset latencies, while support leg condition remained significant. Ankle stiffness was therefore excluded from the remaining analysis.

### Support leg postures

Across all support leg conditions and all muscles, when support leg postures (hip, knee, and ankle angles at trip onset) and support leg condition (control, nonparetic, and paretic) were included in the mixed-effect model, support leg postures were not a significant effect (*p* > 0.30), while support leg condition remained significant. Support leg postures at trip onset are presented in Table 3.

**Table 3:** Stance leg posture

	Stance leg			*p* values		
	Control	Nonparetic	Paretic	Control versus nonparetic	Control versus paretic	Nonparetic versus paretic
Ankle dorsiflexion angle (°)	5.3 ± 1.3	3.3 ± 1.0	−3.7 ± 2.8		0.026	0.053
Knee flexion angle (°)	20.8 ± 1.8	18.5 ± 1.3	5.9 ± 5.5			
Hip flexion angle (°)	25.6 ± 3.2	32.2 ± 2.0	20.8 ± 3.6			0.012
Hip adduction angle (°)	6.2 ± 2.1	1.1 ± 1.3	2.0 ± 1.7	0.012		

Values are intragroup mean ± SE of the mean. Only *p* values that show significant differences between support leg conditions (*p* < 0.05) have been presented. Stance leg joint angles were calculated at trip onset
